# Adipose-Derived Stem Cells as a Tool for Dental Implant Osseointegration: an Experimental Study in the Dog

**Published:** 2015

**Authors:** Eriberto Bressan, Daniele Botticelli, Stefano Sivolella, Franco Bengazi, Riccardo Guazzo, Luca Sbricoli, Sara Ricci, Letizia Ferroni, Chiara Gardin, Joaquin Urbizo Velez, Barbara Zavan

**Affiliations:** 1 *Department of Neurosciences, University of Padua, Padua, Italy.*; 2 *Faculty of Dentistry, University of Medical Science, La Habana, Cuba.*; 3 *Oral Surgery Division, ARDEC, Ariminum Odontologica, Rimini, Italy.*; 4 *Department of Biomedical Sciences, University of Padua, Via Bassi 58, 35100 Padua, Italy.*

**Keywords:** Adipose-derived stem cells, alveolar bone loss, peri-implant bone defect, immediate dental implant, bone regeneration, hydroxyapatite

## Abstract

The biological interaction between the jaw bones and dental implant is fundamental for the long-term success of dental implant placement. Nevertheless, the insufficient bone volume remains a major clinical problem, especially in case of immediate dental implant. Using a canine model, the present study proves the regenerative potential of adipose- derived stem cells (ADSCs) to repair peri-implant bone defects occurring in immediate dental implant placement. In six labradors, all mandibular premolars and the first molars were extracted bilaterally and three months later dental implants were installed with a marginal gap. The marginal defects were filled with hydroxyapatite (HA)-based scaffolds previously seeded with ADSCs. After one month of healing, specimens were prepared for histological and histomorphometric evaluations. Histological analyses of ground sections show that ADSCs significantly increase bone regeneration. Several new vessels, osteoblasts and new bone matrix were detected. By contrast, no inflammatory cells have been revealed. ADSCs could be used to accelerate bone healing in peri- implant defects in case of immediate dental implant placement.

The osseointegration is the direct rigid fixation of the dental implant into jaw bones (1). The long-term success of implant- supported protheses is ensured by the biological interaction between the jaw bones and dental implant surfaces. As implant surface properties strongly influence the bone response, so various techniques of surface treatment have been developped and applied to improve implant stability during bone regeneration (2-4). Nevertheless, a great clinical problem especially in case of immediate dental implant placement is represented by the insufficient bone volume (5). Immediate implant offers several advantages compared to the delayed implant, such as time reduction for making dentures, which may lead to an immediate satisfaction regarding aesthetics and function in patients (6). However, a significant alveolar bone loss is often present as an immediate post-extraction event (5). Several strategies could be applied in this case. The gold standard is represented by autologous bone grafts, but their clinical applications are very limited (7). In this view, the use of stem cells could provide a promising approach for enhancing osseointegration of immediate dental implants, especially for defects in which spontaneous repair is not workable (8-12). Thus, the *in vitro* development of engineered bone tissue offers a hopeful opportunity to repair the peri-implant bone defects.

Bone tissue engineering requires stem cells and an extracellular matrix (ECM) or scaffold, where cells can growth in three dimension. To accelerate the regeneration of bone tissues, grafts need to contain a large number of stem cells or differentiated cells and bioactive factors to attract osteoprogenitor cells to the defect region. In the past years, mesenchymal stem cells (MSCs) isolated from bone marrow (BMSCs) have been used in regenerative medicine, including bone tissue engineering. However, the utility of BMSCs in tissue-engineered bone is restricted due to complicated recovery procedures and limited cell numbers for clinical applications (13-16). Therefore, a different cell source that possesses the same beneficial functions, while simultaneously overcoming their disadvantages, is adipose tissue (17-18). Human adipose tissue can be obtained in larger quantities compared to the more invasive procedure for isolating BMSCs offering moreover little patient discomfort (19-20). Stem cells isolated from adipose tissue are called (ADSCs) and have the same biological properties and characteristics of BMSCs. Indeed, ADSCs express the same cell surface markers and have the same differentiation potentials of BMSCs. Moreover, starting from the same raw material, the quantity of stem cells isolated are bigger from adipose tissue than from other sources, indicating that ADSC could be an important tool for use in regenerative medicine (20-22). In our previous work, we have yet demonstrated the ability of ADSCs seeded onto a hydroxyapatite (HA)-based scaffold to generate an engineered bone tissue. Furthermore, using a rat calvarial defect model, we have demonstrated the osseointegration capability of this HA-based bone graft (23). In this study, we aimed to apply our knowledge in bone tissue engineering in the restoration of peri- implant bone defects occurring in immediate dental implant placement procedures. For this purpose, we have prepared bone substitutes cultivating ADSCs on HA-based scaffolds up to 28 days. Then, we have tested these constructs *in vivo* on a canine model of immediate dental implant placement.

## Materials and methods

**Human ADSCs isolation**

Human ADSCs were extracted from human adipose tissues of five healthy subjects (age: 21-36) undergoing cosmetic surgery procedures, following the guidelines of the university of Padova’s plastic surgery clinic. Written informed consent was obtained from all patients, in accordance with the Helsinki Declaration. The adipose tissues were digested and the cells isolated, expanded and seeded as previously described (18). Briefly, the adipose tissue was washed with phosphate buffered saline (PBS, EuroClone, Milan, Italy) and digested using a solution of 0.075% collagenase from Clostridium histolyticum type II (Sigma-Aldrich, St. Louis, MO, USA) in Hank's balanced salt solution (HBSS, Lonza S.r.l., Milano, Italy), at room temperature under slow agitation for 3 h. The collagenase activity was blocked with an equal volume of cDMEM which consisted of Dulbecco’s modified Eagle’s medium (DMEM, Lonza S.r.l.) supplemented with 10% fetal bovine serum (FBS, Bidachem S.p.A., Milano, Italy) and 1% penicillin/ streptomycin (P/S, EuroClone). After centrifugation for 4 min at 1200 rpm, the pellet was washed in PBS and filtered with a 70 μM cell strainer (BD Biosciences, Mississauga, Ontario, Canada). The cell suspension was suspended in cDMEM, transferred to a 25 cm^2^ tissue culture flask, then incubated at 37 °C and 5% CO_2_. After 3 days, floating cells were discarded and fresh medium was added on the adherent cells. At confluence, ADSCs were harvested by trypsin treatment, then cultivated up to passage 3.

**Biomaterial and ADSCs seeding**

The HA-based scaffolds were supplied in granules (Bio-Oss®, spongious bone substitute, Geistlich Pharma AG, Wolhusen, Switzerland). The granules were coated with 50 mg/mL fibronectin (Sigma-Aldrich) at room temperature for 4 h, then air-dried overnight in a sterile biosafety cabinet. The HA granules were placed in a 1x10^6 ^ADSCs suspension under vacuum conditions for 60 seconds in order to facilitate the cells flow inside the pores. After 2 h of incubation at 37 °C with 5% CO_2_, the scaffolds were cultured in cDMEM up to 28 days, and the medium was changed twice a week.

**MTT assay**

To determine the proliferation rate of cells grown on HA-based scaffolds, the MTT-based (methyl thiazolyl-tetrazolium) cytotoxicity assay was performed according to the method of Denizot and Lang with minor modifications (24). The test is based on mitochondria viability, i.e., only functional mitochondria can oxidize an MTT solution, giving a typical blue-violet end product. After harvesting the culture medium, the cells were incubated for 3 h at 37 °C in 1 mL of 0.5 mg/mL MTT solution prepared in PBS. After removal of the MTT solution by pipette, 0.5 mL of 10% dimethyl sulfoxide in isopropanol was added for 30 min at 37 °C. For each sample, absorbance values at 570 nm were recorded in duplicate on 200 μL aliquots deposited in 96-well plates using a multilabel plate reader (Victor 3, Perkin Elmer, Waltham, MA, USA). All samples were examined

after 7, 14, 21, and 28 days of culture.

**Nuclear staining**

The ADSCs distribution inside the HA-based scaffolds was detected by nuclear staining with Hoechst (H33342, Sigma-Aldrich). The samples were fixed in 4% phosphate- buffered formalin, pH 7, at room temperature for 10 min. After a wash in PBS, cells were stained with 2 μg/mL Hoechst solution in PBS for 5 min.

**Scanning electron microscopy (SEM)**

The HA-based scaffolds seeded with ADSCs were fixed with 2.5% glutaraldehyde in 0.1 M cacodylate buffer at room temperature for 60 min. After a wash in distilled water, samples were dehydrated in graded ethanol and then left in 96% ethanol overnight. Each sample was left at the critical point drying, then covered with a thin layer of gold to make it electrically conductive. All micrographs were obtained at 30 kV on a JEOL 6360LV SEM microscope (JEOL, Tokyo, Japan).

**Real-time PCR**

Total RNA was extracted from each sample by using the TRIzol® Reagent (Invitrogen, Carlsbad, CA, USA). The samples were quantified using the NanoDrop spectrophotometer (Nano Drop™ 1000, Thermo Scientific, Waltham, MA, USA). For the first-strand cDNA synthesis, 500 ng of total RNA was reverse transcribed using M-MLV RT (Moloney Murine Leukemia Virus Reverse Transcriptase, Invitrogen, Paisley, UK) according to the manufacturer's protocol. Human primers were selected for each target gene with Primer 3 software. Real-time PCRs were carried out using the designed primers at a concentration of 300 nM and FastStart SYBR Green Master (Roche Diagnostics, Mannheim, Germany) on a Rotor-Gene 3000 (Corbett Research, Sydney, Australia). Thermal cycling conditions were as follows: 15 min denaturation at 95 °C; followed by 40 cycles of 15 s denaturation at 95 °C; annealing for 30 s at 60 °C; and 20 s elongation at 72 °C. Values were normalized to the expression of the β-actin internal reference, whose abundance did not change under our experimental conditions. Experiments were performed with 3 different cell preparations and repeated at least 3 times.

**Immediate dental implant placement in the canine model**

The research protocol was submitted to and approved by the Ethical Committee of the University of Medical Sciences, School of Dentistry, La Habana, Cuba. 

Six Beagle dogs, with a mean body weight of 11 kg and a mean age of one year, were provided by CENPALAB (Centro Nacional para la Producción de Animales de Laboratorio) of La Habana, Cuba. The surgical procedures were performed at the Centro de Cirugía Experimental (CENCEX) Facultad de Medicina "Victoria de Girón" Universidad de Ciencias Médicas de la Habana (Cuba).

 All the animals were treated and handled in accordance with the “Recommendations for Handling Laboratory Animals for Biomedical Research” compiled by the Committee on the Safe and Ethical Handling Regulation for Laboratory Experiments at the University de l’Havana Cuba. As previously described (25), during surgical procedures the animals were pre-anaesthetized with 0.04 mg/kg atropin plus 0.04 mg/kg medetomidin and 5 mg/kg ketamine, then sedated with isoflurane (1.5% – 3%) in oxygen. All mandibular premolars and the first molars were extracted bilaterally. Three months after the extraction, a crestal incision was performed in the premolar-molar region in both sides of the mandible. Full-thickness mucoperio-steal flaps were elevated, and six experimental sites were identified in the edentulous alveolar ridges, each side of the mandible. The surgical preparation of the sites was performed according to the manual of the implant system (Sweden & Martina, Due Carrare, Padova, Italy). Twist drills were used to prepare each recipient site for implants, 10 mm long and 3.3 mm in diameter (Premium TM, Sweden & Martina). Subsequently, especially designed step drills were used to widen the marginal 5 mm of the implant bed to 5.4 mm. Implants (Premium SP, Sweden & Martina) with 3.3 mm diameter and 10 mm length were subsequently installed with their margin flush to the bone crest and healing caps were screwed on the implants. Following installation, a marginal gap occurred around the implants. The marginal defects in the right side of the mandible were filled with HA-based scaffolds previously seeded with ADSCs. derived from dog’s Bichat bulla, and mechanically dissociated; using previously descri-bed procedures (26). The defects on the left side of the mandible were filled with HA-based scaffolds alone. The flaps were sutured allowing a fully-submerge healing. Animals sacrifice was planned after 1 month of healing. After surgeries, the animals received antibiotic for 8 days (3 ml/48 kg enrofloxacin and 2 mg/kg tramadol). The animals were housed separately in thermostat-controlled cages (22 °C) at the university’s field laboratory with a 12 h day/night cycle, unrestrained with free access to water and fed with moistened balanced dogs’ chow. Postoperatively, the wounds were inspected daily for clinical signs of complications. Check-ups were performed on regular basis throughout the experiment. The animals were euthanatized according to the following protocol: 1000 UI heparin plus 10 mg/kg ketamine, 1mg/kg xylazine, 0.2 mg/kg succinylcholine, and 25 meq KCl.

**Histological processing**

The histological procedures were performed in the laboratory of histology at the Faculty of dontol-ogy of the University of La Habana (Cuba). 

The mandibles were removed, dissected and fixed in 4% phosphate-buffered formalin, pH 7, for 10 days, then transferred to a solution of 70% ethanol until processing. The specimens were dehydrated in increasing concentrations of alcohol up to 100%, infiltrated and embedded in light-polymerized polyester resin (Technovit 7200VLC, Kulzer, Wehrheim, Germany). The specimens were then sectioned along longitudinal axis of the implants using a diamond disc at about 150 mm thick cross section and ground down to about 50-60 microns thickness. Sections were stained with Stevenel’s blue and alizarin red for histo-morphometric analysis using optic microscopy. Immunofluorescence has been performed to track the destiny of ADSC cells. Hoechst staining (Sigma), PCNA (Sigma) and CD31 (Sigma) staining has been used. Briefly, the sections were incubated with the primary antibodies in 2% BSA solution overnight at 4°C in a humidified chamber. The following primary antibodies were used: rabbit polyclonal anti-human CD31 (1∶1000, AB1858, Millipore Corporation, MA, USA), rabbit polyclonal anti- human PCNA antibody (1∶100, ab71822, Abcam). Immuno-fluorescence staining was performed using the secondary antibodies DyLight 549-labeled anti- rabbit IgG (H+L) (KPL, Gaithersburg, MD, USA), DyLight 488-labeled anti-mouse IgG (KPL), and DyLight 549-labeled anti-chicken IgG (H+L) (KPL), which were diluted 1∶1000 in 2% BSA for 1 hour at room temperature. Nuclear staining was performed with 2 µg/ml Hoechst H33342 (Sigma-Aldrich) solution for 2 min.

**Histological assessment**

Histomorphometric analysis was performed on 3 sections from each sample chosen randomly and observed under Leitz Orthoplan light microscope (LM, Leica Microsystem Inc., Bannockburn, IL, USA) equipped with a computerized image analy-zer system (Qwin, Leica Microsystem Imaging Solution Ltd, Cambridge, UK). Five photographs were taken from each section. The standard region of interest (ROI) has been selected in a region of 1218 × 898µm (1.09×106 *μ*m^2^) including porous HA. The pore area inside the HA sample was then examined for growth and bone maturation using the Leica Qwin software. Each sample was analyzed separately (absolute value) regarding the effect of ADSC supplementation or its absence.

**Statistical analyzes**

One-way analysis of variance (ANOVA) was used for data analyzes. The Leveneı´s test was used to demonstrate the equal variances of the variables. Repeated-measures ANOVA with post- hoc analysis using Bonferroni’s multiple comparison was performed. T-tests were used to determine significant differences (P< 0.05). Repeteability was calculated as the standard deviation of the difference between measurements. All statistical analyses were performed in SPSS 16.0 software (SPSS Inc, Chicago, Illinois, USA) (license of the University of Padua, Italy).

## Results

**ADSCs seeding into HA-based scaffolds**

**Fig. 1:**
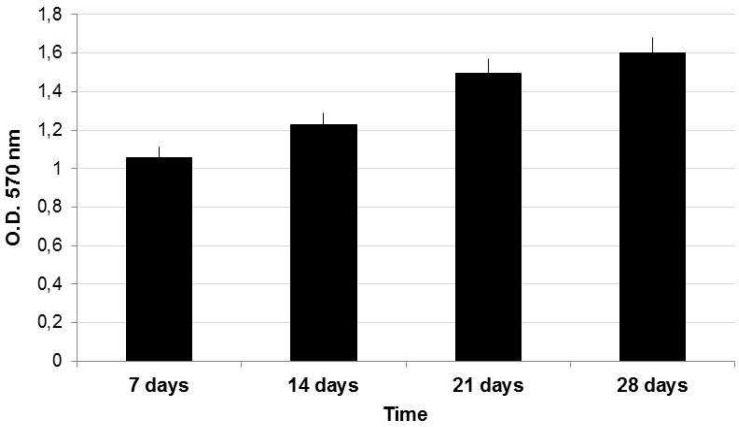
MTT assay of ADSCs cultured onto HA-based scaffolds up to 28 days. The proliferation of ADSCs was evaluated at 7, 14, 21, and 28 days.

**Fig. 2:**
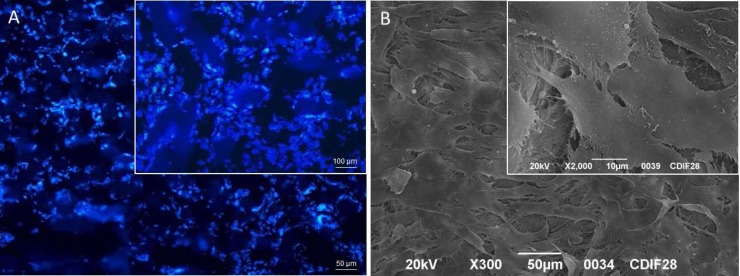
Morphological analyzes of ADSCs cultured into HA-based scaffolds up to 28 days. (**A**) Nuclei of ADSCs seeded into HA-based scaffolds are stained with Hoechst blue fluorescent dye. The cells are attached on surface and into the inner pores of the HA-based scaffold. (**B**) SEM images of ADSCs showing cells with a spindle shape in a continuous layer

**Fig. 3:**
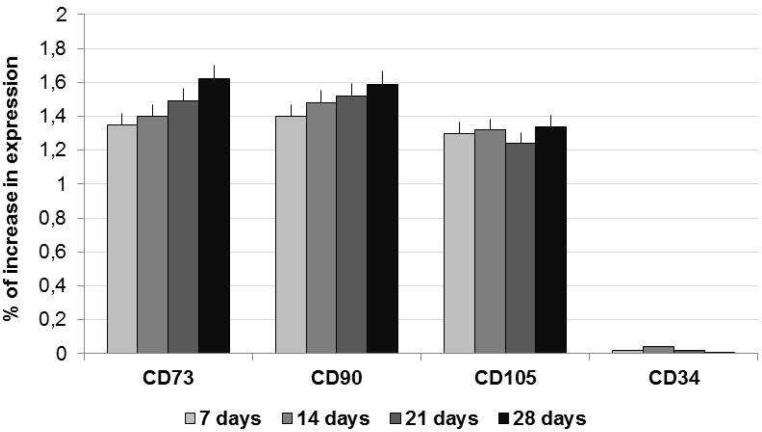
Gene expression profile by means of real-time PCR. ADSCs seeded into HA-based scaffold expressed the MSCs markers CD73, CD90 and CD105, but not CD34 (marker of hematopoietic phenotype

ADSCs amplified in cDMEM up to passage 3 were harvested by trypsin treatment and then seeded into HA-based scaffold. The ADSCs-seeded scaffolds were maintained in cDMEM without any differen-tiation factor in incubator at controlled temperature and carbon dioxide up to 28 days. The proliferation of ADSCs into HA-based scaffold was evaluated by means of the MTT assay performed at 7, 14, 21, and 28 days (Fig. 1). The increasing values registered by MTT assay confirmed the presence of alive ADSCs able to proliferate into the HA-based scaffold during the 28 days of culture. Morphological analyses of the HA-based scaffolds seeded with ADSCs were also performed after 28 days of culture. Nuclear staining with Hoechst, a blue fluorescent dye, showed cells attached on scaffold surfaces and even into inner pores of the substrate (Fig. 2A). This behavior is also observed in SEM images where ADSCs appeared with a spindle shape in a continuous cell layer (Fig. 2B). Stem cells features of ADSCs have been investigated by gene expression profiling with Real-time PCR. The mRNAs of ADSCs seeded into HA-based scaffold in the absence of differen-tiative factors were extracted and molecular markers of MSCs phenotype were investigated. The real-time PCR showed a well-defined expression for CD73, CD90 and CD105, whereas no expression for CD34 has been revealed (Fig. 3).

**Dental implant placement in the canine model**

**Fig. 4:**
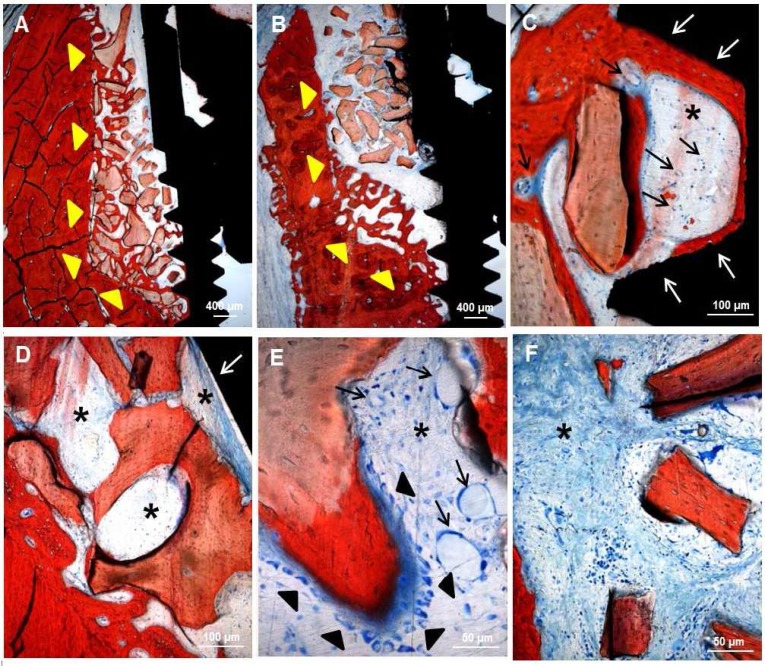
Staining with Stevenel's blue and alizarin red of marginal defects filled with (**A**, **C**, and **E**) HA-based scaffold previously seeded with ADSCs (test condition) or (**B**, **D**, and **F**) HA-based scaffold alone (control condition) after 1 month of healing. In both conditions, a bone regeneration area (yellow arrowhead in **A** and **B**) is present around the implants. At interface between implant and scaffold (white arrows in **C** and **D**) of the test condition new bone tissue (dark red in **C**) is present, whereas a new ECM rich in collagen fibers (asterisk in **D**) is present in control condition. In the defect filled with HA-based scaffold previously seeded with ADSCs several vessels (black arrows in **C** and **E**) embedded in new ECM (asterisk in **C** and **E**) and osteoblastic cells (black arrowhead in **E**) producing new bone matrix (dark red in **E**) are present. In the defect filled with HA-based scaffold alone a new ECM rich in collagen fibers (asterisk in **D **and** F**) is present, but vessels and osteogenic cells are absent.

**Fig. 5:**
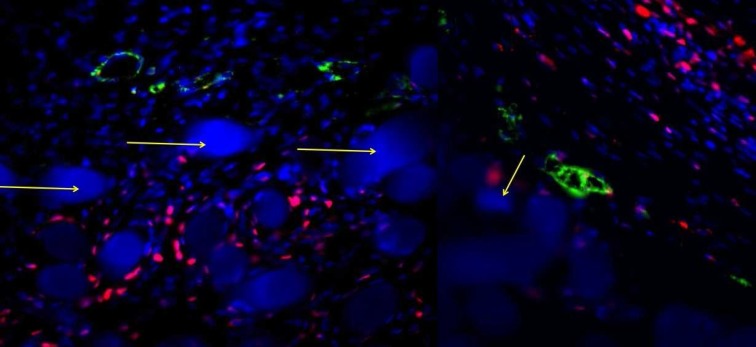
ADSCs destiny analysis by immunofluorescence staining: Nuclei are stained in blue with Hoechst; dog cells are stained as blue point. Around the HA granules, blue stained big points (yellow arrows) are shown

**Fig. 6:**
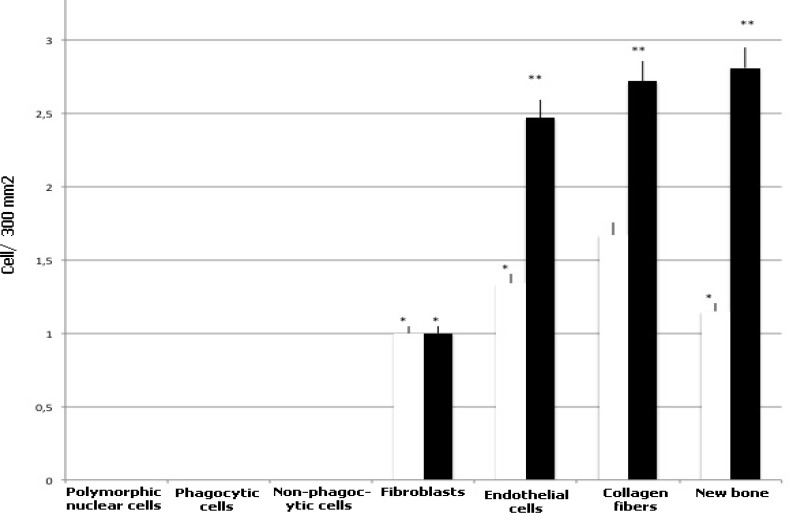
Histological feature of quantitative analyzes of cell population present in the samples treated and not with stem cells. Cells were scored from not present (0) to abundantly present (3). White bars are related to the scaffolds alone and black bars are related to the samples treated with stem cells. Repeated-measures ANOVA with a post-hoc analysis using Bonferroni’s multiple comparison. T tests were used to determine significant differences (P< 0.05). * P< 0.05; * * P< 0.01; * * * P< 0.001. Repeatability was calculated as the standard deviation of the difference between measurements

A canine model has been employed to assess the bone regeneration activity of ADSCs. Six dental implants were installed in the alveolar ridges on each side of mandible with a marginal gap around the implants. The marginal defects in the right side of the mandible were filled with HA-based scaffolds previously seeded with ADSCs up to 28 days (test condition). Conversely, the defects in the left side of the mandible were filled with HA-based scaffolds alone (control condition). Histological analyses of ground sections stained with Stevenel's blue and alizarin red revealed in both conditions the occurrence of bone regeneration around the implants after 1 month of healing (area indicated by yellow arrowheads: test condition in Fig. 4A; control condition in Fig. 4B). In both conditions, new ECM rich in collagen fibers has been deposited (black asterisks in Fig. 4C and Fig. 4D). In particular, the HA-based scaffold enriched with ADSCs showed the presence of several vessels (black arrows in Fig 4C and Fig. 4E) embedded in the new ECM; on the contrary, vessels could not be detected in the HA-based scaffold alone (Fig.4D and Fig. 4F). In the test condition, a regenerated bone tissue (dark red in Fig. 4C) was present at interface between implant and HA-based scaffold (white arrows in Fig. 4C). Presumably, the presence of ADSCs has considerably stimulated the osteogenic population (black arrowheads in Fig. 4E) mainly around the HA-based scaffolds (light red in Fig. 4E) to produce new bone matrix (dark red in Fig. 4E). Conversely, in the control condition, an ECM of collagen fibers (black asterisks in Fig 4D) was lay down at interface between implant and HA-based scaffold (white arrows in Fig. 4D), however, osteogenic cells were not detected (Fig. 4F). The destiny of ADSCs has been followed by immunofluorescence analysis. Results reported on fig. 5 revealed that all samples were highly populated by dog cells as blue point (related to the blue staining of the nuclei with Hoechst) revealed. Around the HA granules also in this case, blue stained big points (yellow arrows) are shown. Around the HA samples, a rime of cells stained in red is well evident. These cells are human ADSCs positive for PCNA, a specific antibody for human nuclei. In this way, human stem cells were marked and we found that inside a dog population (blue nuclei), after 1 month, human ADSCs (nuclei stained in red) are still present,. The presence of cells with red nuclei around the HA scaffolds confirm the involvement of ADSCs in the new bone formation (NBF) process. Interestingly, the vessels present in the tissue (stained in green, thanks to the positivity of CD31 marker which is specific to endothelial cells) show both blue and red nuclei establishing that ADSCs participate also to the new vascularization process. No sign of inflammatory process was detected after thirty days after surgery (Table 1 and fig. 6). In both conditions, polymor-phic nuclear cells (i.e. granulocytes), phagocytic cells (including macro-phages and monocyte-derived giant cells), and non-phagocytic cells (comprising lymphocytes, plasma cells, and mast cells) were absent. The defects filled with HA-based scaffolds alone (white bars) showed a scarce amount of fibroblasts, endothelial cells, collagen fibers and NBF. By contrast, the defects filled with HA-based scaffolds previously seeded with ADSCs (black bars) showed a larger amount of endothelial cells and collagen fibers. In addition, the formation of new bone is more evident. Histomorphological analyses related to the NBF has been performed and reported on Table 2. Evidently, the bigger values on NBF are in the site on which ADSCs are present.

## Discussion

Often, the installation of dental implants is hampered by alveolar bone loss, especially in immediate implant placement. In order to reduce the time of the bone healing around the implant and induce better osseointegration of the implant, several studies were performed in the last decade (27). Much attention was paid to changing the shape of implants and modifying the surface properties of the dental implants, but the develop-ment of engineered bone tissue could offer a new tool for peri-implant bone defect repair. The bone healing process around the implant is a biolo-gical event strongly similar to bone healing process involved after a bone injury repair and involves several important steps such as immune system activation, angiogenesis, osteo-genesis. Moreover, tooth extraction and the following dental implant position induces an acute injury, recruiting MSCs derived from wound site to contribute to the process of bone regeneration (28-30).

**Table 1:** Histomorphometric analysis of defects filled with HA-based scaffolds alone or previously seeded with ADSCs for 28 days

	**HA-based scaffolds alone**	**HA-based scaffolds with ADSCs**
Polymorphic nuclear cells^a^	**-**	**-**
Phagocytic cells^b^	**-**	**-**
Non-phagocytic cells^c^	**-**	**-**
Fibroblasts	**+**	**+**
Endothelial cells	**+**	**++**
Collagen fibers	**+**	**++**
New bone	**+**	**++**

Cells were scored from not present (-) to abundantly present (+++).

a Polymorphic nuclear cells include i.e. granulocytes;

b Phagocytic cells include macrophages and monocyte- derived giant cells;

c Non-phagocytic cells include lymphocytes, plasma cells and mast cells.

**Table 2:** Histomorphological analyses related to the NBF

**DOG**	**NBF in presence of ADSCs**	**NBF in absence of ADSCs**
1	146937/μm^2^	96542/μm^2^
2	152985/μm^2^	109007/μm^2^
3	135982/μm^2^	105874/μm^2^
4	112345/μm^2^	98765/μm^2^
5	142367/μm^2^	97623/μm^2^
6	135987/μm^2^	119002/μm^2^

First studies on MSCs were carried out in 1960s by Friedenstein et al. who found that the bone marrow is MSCs' reservoir. Moreover, for the first time they characterized them by observing that these cells are plastic-adherent, have fibroblast-like morphological feature and can be committed into chondrocytes, adipocytes and osteoblasts. Afterw-ards, several research has demontrated that MSC treatment could be very useful for bone repair (31).

During this process, osteoinductive molecules are involved to support the cellular migration and differentiation of the new progenitor cells required for the bone helaing (32). In this context, MSCs are one of the most important cell types influenced by these osteoinductive molecules (33). Their important role on bone regeneration process makes them an important tool to encourage tissue repair process primarily in cases of lack of adequate supply of autologous bone graft or unsuitable allograft use. Moreover, MSCs show also immunosuppressive behavior by suppressing the proliferation of T-cells in the presence of alloantigens and inhibiting the function of B cells (34). Thank to these interesting properties and encouraging research results the bone regeneration process supported by MSCs is becoming clinicaly possible (35).

Much has already been done in the field of regenerative medicine, where the use of stem cells has allowed the regeneration of different tissues, including the bone tissue. Several studies related to the application of stem cells applied to bone defect repair showed that MSCs can fill the defect region producing novel extracellular matrix component and cytokine ensuring quick good therapeutic effects (36).

In light of such considerations, we have tested the hypothesis that stem cells isolated from adipose tissue can promote osseointegration between dental implants and engineered bone tissue. It has been widely demonstrated that ADSCs are multipotent stem cells with the capacity to differentiate into different lineages, such as bone, cartilage, fat and endothelial cells (37). These features make ADSCs the ideal cells to apply in regenerative medicine by means of tissue engineering strategies. To reach this purpose, we have implanted HA-based scaffolds enriched with ADSCs in alveolar bone defects during procedures of immediate dental implant placement in canine models.

Our results showed that ADSCs are able to adhere and proliferate into the HA-based scaffold, and to preserve the MSCs features. Once HA-based scaffolds enriched with ADSCs were inserted into marginal defect around dental implant, several new vessels and new bone matrix were detected. By contrast, no inflammatory cells have been revealed. Thus, bone regeneration process observed in our experimental data, is mediated by the transplanted ADSCs that may act by starting to recruit endogenous stem cell by means of trophic factor secretion without triggering any inflammatory response (38). In conclu-sion, our HA-based scaffold enriched with ADSCs could be a feasible and effective engineered bone tissue to use to accelerate bone healing in peri-implant defects.
